# Ultrasound-mediated piezoelectric differentiation of neuron-like PC12 cells on PVDF membranes

**DOI:** 10.1038/s41598-017-03992-3

**Published:** 2017-06-22

**Authors:** Marcus Hoop, Xiang-Zhong Chen, Aldo Ferrari, Fajer Mushtaq, Gagik Ghazaryan, Theo Tervoort, Dimos Poulikakos, Bradley Nelson, Salvador Pané

**Affiliations:** 1 Multi-Scale Robotics Lab (MSRL), Institute of Robotics & Intelligent Systems (IRIS), ETH Zurich, Zurich, 8092 Switzerland; 2 Laboratory of Thermodynamics in Emerging Technologies, ETH Zurich, Zurich, 8092 Switzerland; 3 Department of Materials, ETH Zurich, Zurich, 8093 Switzerland; 4 Laboratory for Biointerfaces, Empa, St. Gallen, 9014 Switzerland

## Abstract

Electrical and/or electromechanical stimulation has been shown to play a significant role in regenerating various functionalities in soft tissues, such as tendons, muscles, and nerves. In this work, we investigate the piezoelectric polymer polyvinylidene fluoride (PVDF) as a potential substrate for wireless neuronal differentiation. Piezoelectric PVDF enables generation of electrical charges on its surface upon acoustic stimulation, inducing neuritogenesis of PC12 cells. We demonstrate that the effect of pure piezoelectric stimulation on neurite generation in PC12 cells is comparable to the ones induced by neuronal growth factor (NGF). In inhibitor experiments, our results indicate that dynamic stimulation of PVDF by ultrasonic (US) waves activates calcium channels, thus inducing the generation of neurites via a cyclic adenosine monophosphate (cAMP)-dependent pathway. This mechanism is independent from the well-studied NGF induced mitogen-activated protein kinases/extracellular signal-regulated kinases (MAPK/ERK) pathway. The use of US, in combination with piezoelectric polymers, is advantageous since focused power transmission can occur deep into biological tissues, which holds great promise for the development of non-invasive neuroregenerative devices.

## Introduction

Neurotrauma and neurodegenerative diseases, such as Alzheimer’s and Parkinson’s, have devastating effects on the life of more than 30 million people worldwide^1–3^. In general, these diseases result in irreversible structural disruption of the neuronal network accompanied by cell death^4, 5^. Unfortunately, adults have limited capability to actively regenerate or replace neuronal tissue^6^. A landmark study in the field of neurobiology by Richardson *et al*. demonstrated that the deficiency of neuronal regeneration is not an intrinsic cell property, but is rather caused by an unfavourable growth environment within the damaged tissue^7^. As a consequence, if neurons are provided with the correct set of biological and physical stimuli, regeneration of the damaged neuronal tissue can occur^8^. The most prominent group of neuronal stimulation factors used today are neurotrophins, such as nerve growth factor (NGF), inducing intracellular pathways that stimulate cell proliferation, survival, and differentiation^9^. Incorporation of neurotrophins, e.g. NGF, into polymer scaffold has been examined for efficient nerve repair stimulation *in vitro* ^10, 11^. However, the short half-life time and fast diffusion of NGF, significantly limits their efficiency *in vivo* ^12^.

As an alternative to conventional NGF treatments, studies have demonstrated the potential of electrical charges for the differentiation of multiple cell types^13^ including neurons^14, 15^. Enhanced neuronal outgrowth has been shown on conductive materials, such as polypyrrole, carbon nanotubes and indium tin oxide under electrostimulating conditions^16–19^. However, applicability of these materials in clinical devices has been hampered by the necessity of using wired electrical stimulation with external power supplies, which makes these devices invasive, causing chronic wounds with increased risk of infection and inflamation *in vivo*. Therefore, identifying new electroactive scaffold materials for enhanced wireless neuroregeneration remains a major challenge for neuronal biomedicine.

Piezoelectric materials, which can generate transient charges on their surfaces upon mechanical stimulation, have attracted increasing attention because of their potential application for remote control, regenerative therapy^20, 21^. They have been used as piezoelectric nanoparticles, such as boron nitride and barium titanate, to demonstrate enhanced neurite outgrowth under ultrasound (US) stimulation in neuronal-like and neuroblastoma-derived cells^22, 23^. To make use of this unique feature, these nanoparticles were embedded into polymer nanocomposites through two photon lithography or into hydrogels to fabricate scaffolds^24, 25^. Piezoelectric polymers, e.g. PVDF and its copolymers, have also been successfully used in this context. Such polymeric materials, which possess a superior processability compatible with current scaffold shaping techniques, can easily be used to fabricate piezoelectric scaffolds. Arinzeh’s group demonstrated the feasibility of using electrospun piezoelectric P(VDF-TrFE) scaffolds for enhanced proliferation and stimulation of stem and neuron cells^26–28^. Nevertheless, those studies were conducted under static conditions where the influence of the piezoelectric P(VDF-TrFE) property on the cell fate under dynamic stimulation was not investigated. The suitability of polymer/ceramic piezoelectric composite films under US for neuronal stimulation through direct piezoelectric effect was demonstrated by Genchi *et al*. using P(VDF-TrFE) and BaTiO_3_^29^. Besides, a recent work from Lanceros-Mendez group demonstrated wireless stimulation of cells using P(VDF-TrFE)/Terfenol-D composite film through magnetoelectric coupling effect^30^.

**Figure 1:**
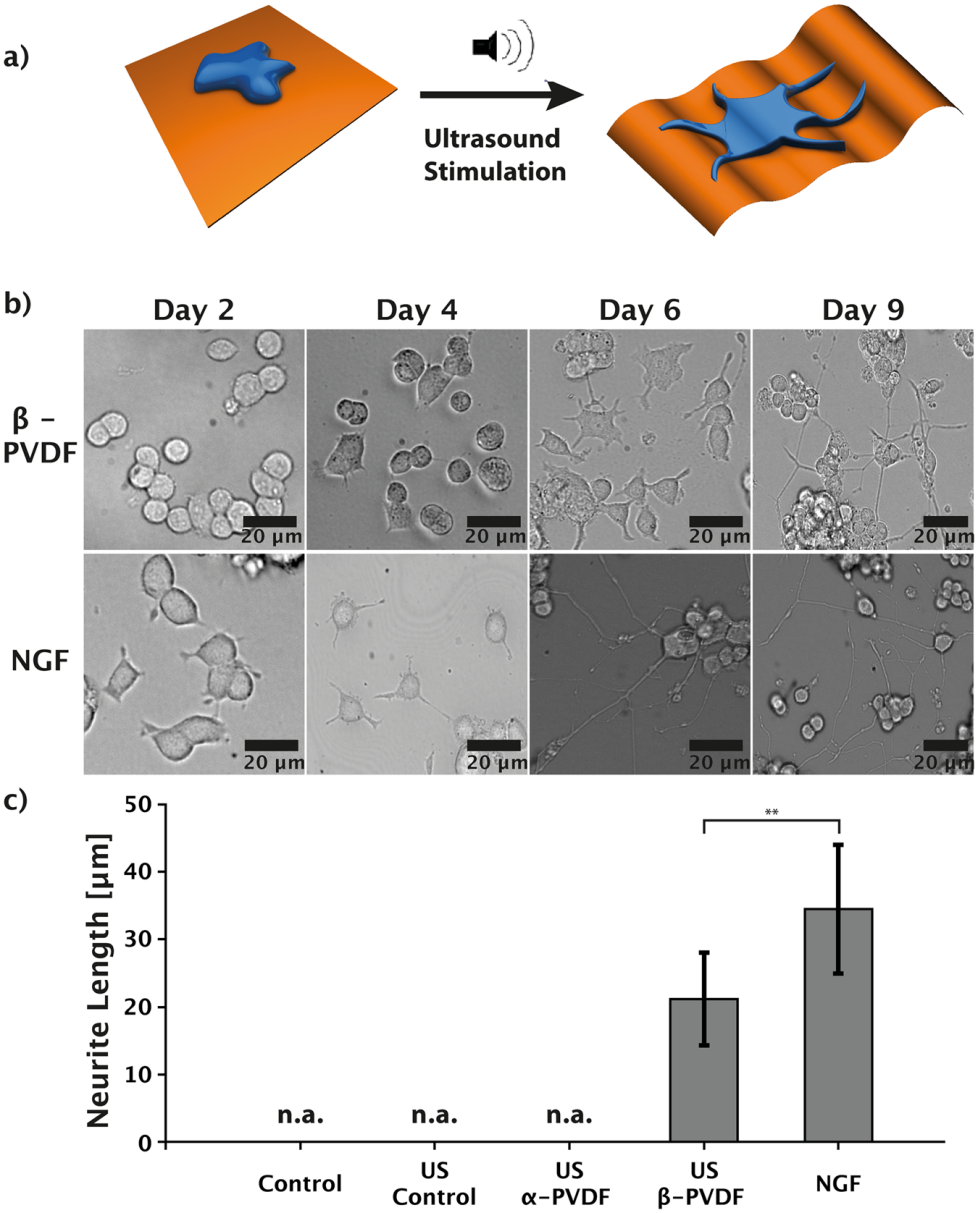
(**a**) Schematic illustration: Ultrasound (US) stimulation of the piezoelectric β-PVDF membrane induces neuronal differentiation of PC12 cells due to wireless, mechanical deformation of the β-PVDF membrane. (**b**) Time-lapse images of PC12 cells stimulated by NGF or piezoelectric β-PVDF upon US stimulation. (**c**) Comparison of average neurite length of PC12 cells cultured in media (control), media with US (US control), cultured on non-piezoelectric α-PVDF with US stimulation, cultured on piezoelectric β-PVDF with US stimulation, and cultured with NGF. **p < 0.01; n.a.: measured protrusions lower than 10 µm – not considered as neurites.

In this work, we investigate the role of the piezoelectric polymer β-polyvinylidenfluorid (β-PVDF) as a candidate to support neuronal differentiation. The piezoelectric properties of β-PVDF allow for the generation of electrical charges on its surface as a result of mechanical deformation^31^. Compared with P(VDF-TrFE), which is piezoelectric in any case, the non-piezoelectric α-PVDF provides a counterpart that allows for ruling out the influence of the surface chemistry and/or mechanical factors on the induction of PC12 differentiation using β-PVDF. Besides, PVDF is more accessible due to its low cost and high production volume. By using the PC12 model for neuronal differentiation of mammalian cells^32^, we examine the capability of the piezoelectric surface characteristic for the initiation and growth of cell neurites (Fig. 1a). Ultrasound (US) is utilized as a power transmission system for wireless dynamic stimulation of β-PVDF. US has been widely employed in biomedical devices due to its superior biocompatibility and deeply focused penetration in biological tissues^33^. The electrical charge generation by β-PVDF is focused solely around the polymer, allowing for enhanced spatial control of the neuronal stimulation area. In addition, the molecular differentiation pathway induced by the electrostimulation of PC12 cells is investigated through dedicated experiments with biochemical inhibitors specifically targeting alternative differentiation pathways. The capability of US stimulated β-PVDF membranes to induce neuronal differentiation is non-invasive and allows for spatially precise, long-term therapy, making it an ideal candidate for the development of novel neuroregenerative devices.

## Results and Discussion

### *In vitro* neuronal-like cell stimulation by piezoelectric PVDF

To promote cell adhesion, β-PVDF membranes were pre-treated with poly-L-lysine. In the stimulation experiment, PC12 cells cultured on the piezoelectric β-PVDF membranes were exposed to US for 10 minutes, five times per day. Negative control experiments, where cells were cultured with or without US stimulation on β-PVDF, α-PVDF, or directly on well plates coated with PLL were carried out. The phase of β-PVDF and α-PVDF is almost pure according to the calculation using the method reported in literature^34^ (see Supplementary Fig. S1). Positive control experiments (NGF stimulation) were also performed for comparison. Further experimental details can be found in the experimental section.

Initially, PC12 cells were seeded on pre-treated β-PVDF membranes or on normal tissue-culture plates with NGF. Phase-contrast optical microscope images (Fig. 1b) after two days of US stimulation showed no morphological change in PC12 cells on the piezoelectric β-PVDF membrane. First protrusion formation of piezoelectrically stimulated PC12 cells was observed at day 4, and continuous exposure to the US led to further growth of the neurite length. Maximum neurite outgrowth was reached at day 9 (see Supplementary Fig. S2). In comparison, PC12 cells stimulated with NGF rapidly showed small protrusion formation after day 2, and continued to increase in length until day 6. Continued stimulation until day 9 did not induce further neurite outgrowth.

Figure 1c shows the average neurite length measure in PC12 cells cultured under different conditions after nine days of stimulation. Here, only the cells with neurites longer than 10 μm are considered as differentiated^35^. US treatment of PC12 cells on a piezoelectric β-PVDF membrane induced differentiation with an average neurite outgrowth of 22.9 μm ± 6.8 μm. PC12 cells subjected to NGF stimulation showed neurite formation with an average length of 34.5 μm ± 9.5 μm. In contrast, unstimulated PC12 cells and cells stimulated only by US (without a PVDF substrate) showed only small protrusions (average length 2.3 μm ± 1.8 μm and 3.1 μm ± 1.5 μm, respectively) and no formation of neurites. Cells cultured on non-piezoelectric α-PVDF with US stimulation also showed no neurite formation (average protrusion length 4.6 μm ± 3.1 μm). Typical cell morphology in these control experiments are shown in Supplementary Fig. S3.

These results indicate that β-PVDF membranes are able to induce neuronal differentiation with comparable efficiency to commonly used NGF protocols. Control samples (PC12 on tissue culture plates with and without US stimulation) showed short membrane protrusions but no genuine neurites^36^. From these results, we can further conclude that the stimulation offered by US alone has no significant influence on PC12 differentiation.

It has been reported that surface and mechanical properties of substrates can also affect cell dynamics^37, 38^. In order to exclude the influence of surface/mechanical factors on the induction of PC12 differentiation, non-piezoelectric α-PVDF sheets (same surface chemistry/similar mechanical properties as β-PVDF) were used for control experiments. Also the crystallinity of the α- and β-PVDF films used in this study are very similar, with measured values of 44.3% and 50.0%, respectively (Supplementary Fig. S4). The Young’s modulus of α-PVDF is slightly lower than that of β-PVDF but they are still of the same magnitude (~GPa, Table S1). In any case, the moduli of both films are much higher than the moduli range (10 Pa ~750 kPa)^39^ in which neuronal cells are extensively studied regarding their mechanoconduction behavior. On substrates with Young’s modulus in the GPa range, the cell ability to deform the substrate and thus sense gradients of stiffness is well beyond saturation. To our knowledge, no previous work describes the mechanoconduction behavior of PC12 cells on such stiff substrates. However, it has been found that decreased substrate rigidity in the range of kPa~MPa leads to an increase in neurite branching and extension^40, 41^. In our case, α-PVDF has a lower modulus compared to β-PVDF. Therefore, differences in mechanical properties cannot be the main reason for promoting cell differentiation. We also investigated the surface morphology of both films (Supplementary Fig. S5). The root mean square surface roughness of the α-PVDF films (7.418 nm) is somewhat smaller than that of the β-PVDF films (11.807 nm), indicating a slightly smoother surface. However, according to previous studies^42^, neither neurite number per cell nor neurite length is affected by surface roughness when it varies from 3.5 to 16 nm. Therefore, significant differences in the induction of neurite formation between α- and β-PVDF under US stimulation clearly indicate that differentiation is caused by the piezoelectric properties of β-PVDF. This result is consistent with recent studies conducted by Lanceros-Mendez’s group, where the proliferation and differentiation of osteogenic cells were promoted by piezoelectric PVDF exposure to dynamic mechanical stimulation^43, 44^. The advantageous feature of wireless deep tissue penetration in the human body makes ultrasound an attractive choice over direct mechanical transduction for *in vivo* medical applications.

### Mechanism of neuronal differentiation induced by piezoelectric stimulation

**Figure 2:**
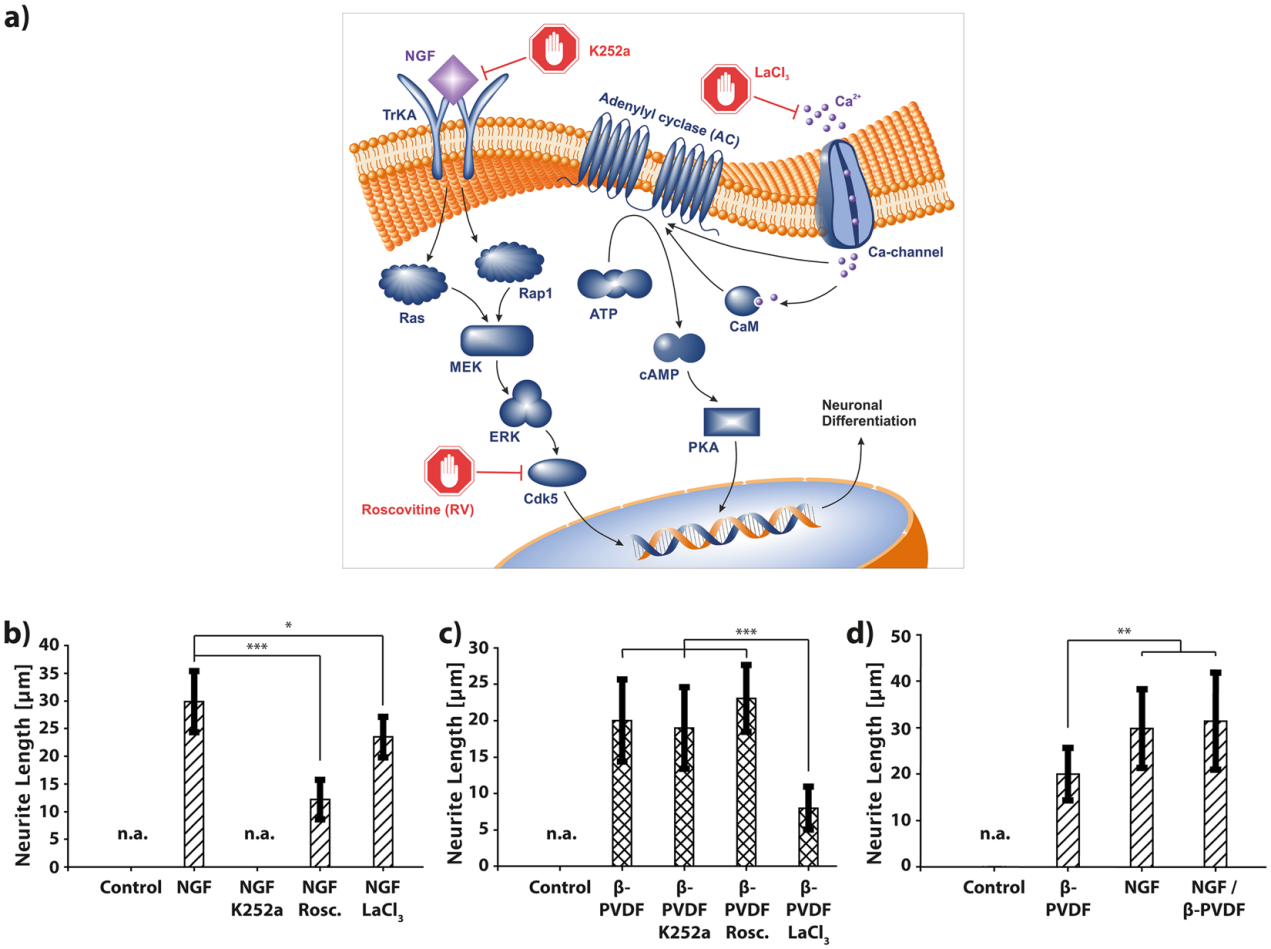
(**a**) Illustration of intracellular pathways affecting PC12 differentiation. On the left, the well-studied TrKA/Ras/MEK/ERK pathway. On the right, the proposed differentiation pathway induced by piezoelectric depolarization of the PC12 cell membrane. (**b**,**c**) Inhibitor experiments, showing average neurite length of PC12 cells stimulated by β-PVDF and US or NGF under the influence of the inhibitors K252a, LaCl_3_, and Roscovitine (RV). (**d**) Comparison of the stimulation effect of β-PVDF, NGF, and β-PVDF and NGF on the differentiation of PC12 cells. ***p < 0.001; n.a.: measured protrusions lower than 10 µm – not considered as neurites.

Inhibitor experiments were conducted to investigate the underlying molecular mechanism of the PC12 differentiation by piezoelectric β-PVDF stimulation. Figure 2a illustrates the two different molecular signaling pathways of PC12 differentiation: the MAPK/ERK (mitogen-activated protein kinases/extracellular signal-regulated kinases) pathway and the cAMP (cyclic adenosine monophosphate) – dependent pathway^45, 46^. It is known that NGF induced differentiation relies on the MAPK/ERK pathway. NGF molecules bind to tyrosine kinase receptors (TrkA), causing intracellular phosphorylation. Consequently, the activation of Ras/Rap1 initiates the downstream signaling cascade MEK→ERK→Cdk5, promoting PC12 neuronal differentiation. Another pathway, i.e. cAMP-dependent pathway, also known as the adenylyl cyclase (AC) pathway, can be triggered by a variety of extracellular stimuli. For example, it has been demonstrated that an elevated intracellular concentration of calcium ions (Ca^2+^) leads to the activation of adenylyl cyclase (AC) either directly or indirectly via complex formation with calmodulin^47^. As a key regulatory enzyme, AC catalyzes the conversion of ATP to cyclic AMP (cAMP)^48^, a secondary messenger molecule that is responsible for multiple regulatory signaling pathways, such as the activation of the protein kinase A (PKA) dependent neuronal differentiation pathway^46^. Previous studies have shown that electrical pulses can activate calcium channels in cells, resulting in an increased cellular Ca^2+^ influx^49, 50^. Therefore, it is likely that the observed PC12 differentiation upon piezoelectric stimulation happens via the AC signalling cascade.

To test our hypothesis, PC12 cells were treated with different inhibitors such as K252a, roscovitine (RV), and lanthanum chloride (LaCl_3_). K252a can block the TrkA receptor^51^, and RV can block p35/cyclin dependent kinase (Cdk5)^52^. They are both known inhibitors for neuronal differentiation via the NGF triggered differentiation pathways. LaCl_3_ was used as a Ca^2+^ channel blocker in order to investigate the possible effect of piezoelectric stimulation on the activation of Ca^2+^ channels^53^. Figure 2b, shows that the average neurite length of NGF stimulated PC12 cells was significantly inhibited in the case of RV and K252a due to the inhibition of Cdk5 or TrkA, respectively. K252a inhibition almost completely suppressed the PC12 differentiation, whereas RV primarily reduced the neurite growth. The use of LaCl_3_ showed only a minor influence on the NGF dependent differentiation effect. For piezoelectric-dependent PC12 differentiation, an opposite inhibition scheme was observed (Fig. 2c). Here, the TrkA and Cdk5 inhibitors of the NGF signaling pathway, i.e. K252a and RV did not show any influence on the development and growth of the neurites. However, using the Ca^2+^ channel blocker, LaCl_3_, caused a significant reduction of neurite outgrowth, comparable to the inhibition observed for K252a and RV for the NGF stimulation experiments. These results suggest that the piezoelectric stimulation causes the activation of Ca^2+^ channel, and the increased Ca^2+^ influx plays a substantial role in the differentiation of PC12 cells on piezoelectric β-PVDF. Furthermore, the inhibitor experiments suggest that both mechanisms of piezoelectric β-PVDF and NGF dependent differentiation are independent from each other, since NGF inhibitors did not show any influence on β-PVDF stimulation and vice versa.

To further elucidate the independent action of NGF and piezoelectric stimuli, we investigated simultaneous stimulation of PC12 cells by both NGF and piezoelectric β-PVDF. Figure 2d, shows the average neurite length for PC12 cells stimulated by β-PVDF, by NGF, and NGF/β-PVDF. The results show that simultaneous stimulation of PC12 by NGF and piezoelectric β-PVDF did not significantly increase neurite outgrowth. These results indicate that both stimulation mechanisms are independent and mutual activation does not affect differentiation efficiency.

### Polarization

**Figure 3:**
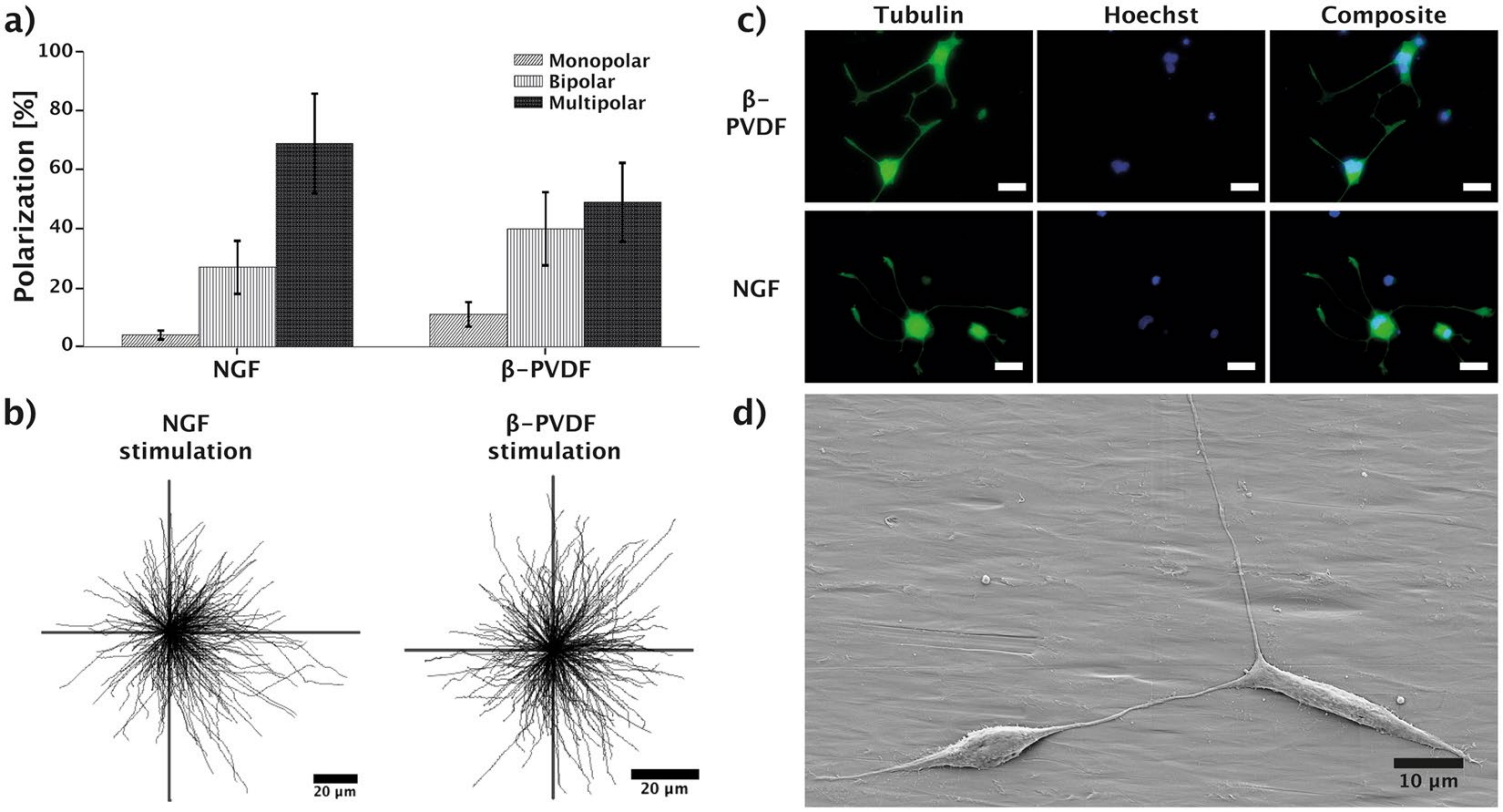
(**a**) Polarization of differentiated PC12 cells stimulated by NGF or β-PVDF/US. (**b**) Tracing of the measured neurites, showing their length and directionality. (**c**) Fluorescence images of stained tubulin (green) and nucleus (blue), differentiated PC12 cells stimulated by β-PVDF/US or NGF; scale bar represents 10 μm. (**d**) SEM image showing differentiated PC12 cells on the β-PVDF membrane after nine days of US stimulation.

The polarization of neuron cells characterizes the formation of axons and dendrites in neuron cells and is crucial for neuronal functionality^54, 55^. Many extra – and intracellular processes are responsible for the establishment and maintenance of cell polarity, such as protein diffusion and scaffold formation, secretory pathways, and ion channel activity^56^. To assess the influence of piezoelectric stimulation on the polarity of PC12 cells, we investigated the polarity distribution of stimulated PC12 cells on β-PVDF and under NGF stimulation. Figure 3a shows that NGF induced differentiation leads to a higher formation of multipolar cells (69%) compared to β-PVDF (48%) stimulated cells. Formation of bipolar (27%) and monopolar (4%) cells was rarely observed for NGF stimulated cells, whereas piezoelectric stimulation showed an increase in the formation of bipolar cells (40%). Figure 3b provides an overview of the neurite length and orientation of all investigated PC 12 cells stimulated by NGF and PVDF. The results confirm that the stimulation with PVDF is isotropic and does not provide a preferential direction to neurite initiation or outgrowth, although the surface of the β-PVDF shows some aligned grooves (Supplementary Fig. S5). Several investigations have evaluated the influence of topographic cues on neurite growth. The majority of these studies used microgrooves with depths ranging from 200 nm to 69 μm^57^. While the grooves used in previous studies usually have well-defined shapes, i.e., square cross section with sharp edges, the aligned polymer structures on our β-PVDF films are much less ordered and exhibit small heights (<50 nm, Supplementary Fig. S5). Previous reports demonstrated that adherent eukaryotic cells can sense and respond to gratings with vertical size down to 35 nm. Shallower topographies are equivalent to flat surfaces in terms of contact guidance^58^. In addition, it has been proven that noisy nanotopographies with specific directionality are not able to induce a preferential neurite extension^59^. These reports may explain why neurite guided extension was not observed on our β-PVDF films. Such stimulation has the potential to be coupled with substrates inducing directionality in neurite growth by means of topography^60^ or gradients of rigidity or adhesion points^61^. Fluorescence images (Fig. 3c) further confirm this observation on cell polarity, where cells stimulated on PVDF showed a lower degree of neurite formation compared to NGF treated PC12 cells. Similar results can be seen in the SEM image (Fig. 3d) of two interconnected and differentiated PC12 cells stimulated on β-PVDF having a bipolar morphology.

## Conclusions

Inducing differentiation of neuronal cells by wirelessly stimulated piezoelectric polymers can create a new therapeutic avenue for contactless, controlled neuroregenerative therapies. As a proof of concept, we provide an *in vitro* analysis on the application of the piezoelectric polymer β-PVDF for the initiation of PC12 cell differentiation. The results show that externally applied US waves are sufficient to induce polarization in piezoelectric polymer sheets, hence, inducing differentiation of PC12 cells. The differentiation efficiency is comparable to conventional *in vitro* differentiation protocols using NGF. The neurite outgrowth was uniform in all directions, which suggests that the electrical stimulus by the β-PVDF membrane induces an overall differentiation pressure without directional neurite extension. Additionally, inhibitor experiments elucidate the underlying molecular pathway responsible for piezoelectric β-PVDF induced differentiation, which has been shown to be independent from the well-known NGF induction process. Our results indicate that the electrical polarization of the β-PVDF membrane induces a repolarization of the cell membrane, resulting in an enhanced influx of Ca^2+^. Subsequently, downstream mechanisms cause the activation of the cAMP/PKA pathway, which leads to the induced differentiation potential of PC12 cells. The results obtained in this study indicate that piezoelectric β-PVDF can be fabricated and integrated into cost-effective scaffolds and devices that can be stimulated by US for minimally invasive neuronal regeneration. In addition, the polymeric material used in this study allows for facile doping with drugs or neuronal growth factors for enhanced tissue regeneration by a multi stimulus system.

## Materials and Methods

### Materials and characterization

Poled β-PVDF membrane was purchased from Precision Acoustics Ltd, UK. The piezoelectric coefficient d_33_ is −30 ± 2 pC/N. α-PVDF membrane was purchased from CS Hyde. Both β-PVDF and α-PVDF membranes have a thickness of 25~30 μm. The crystalline phase was confirmed by X-Ray diffraction pattern (Bruker D8 Advance). Infrared spectra were measured using a VERTEX 70 (Bruker, Germany) Fourier transformed infrared spectrometer equipped with a silicon ATR crystal. Spectra were recorded in the range of 4000 ~ 400 cm^−1^ at 4 cm^−1^ resolution averaged from 64 scans. Thermal analysis was conducted using a differential scanning calorimeter (DSC 822^*e*^, Mettler Toledo, Switzerland) calibrated with Indium for temperature and enthalpy. DSC thermograms were recorded under N_2_ purge at standard heating and cooling rates of 10 °C min^−1^, using a sample weight of ca. 5 mg. Tensile test measurements of the PVDF films were performed on a static mechanical tester (Instron^®^ 5864, USA) fitted with a 0.1 kN load cell. Dumbbell-shaped specimens with a gauge length and width of 12 mm and 2 mm (ISO 527-2), respectively, were cut out and tested at 37 °C. The value of the Young’s modulus, refers to an average value of at least three measurements.

To measure the voltage generated upon ultrasonic stimulation, the membrane was coated with 100 nm gold on both sides using thermal evaporation. A copper wire was attached to the gold coating on each side to provide connection with an oscilloscope (LeCroy WJ324). The membrane was covered with a thin layer of polystyrene by dip-coating in polystyrene/toluene solution (2% w/v) and the copper wires are sealed with kapton tape in order to prevent short-circuit. A photograph of the device and a typical output voltage profile is shown in Supplementary Fig. S6.

### Sample preparation

The PVDF membrane was cut into 1 cm^2^ sheets. The sheets were placed three times for 10 min in 70% ethanol. Afterwards, films were air dried and exposed to ultraviolet light (UV) for 20 min.

The PVDF sheets were placed in a 24 well-plate and incubated with poly-l-lysine (PLL) solution overnight. Then, the PLL was removed and the sheets were dried for 1 h at room temperature. Finally, the sheets were washed with PBS.

### Cell culture

PC12 cells were grown in RPMI medium supplemented with 10% horse serum (HS), 5% fetal bovine serum (FBS), 2 mM glutamine, and 1 x antibiotic antimycotic solution and were maintained at 37 °C, and 5% CO_2_.

For piezoelectric stimulation experiments, PC12 cells were harvested and transferred to the differentiation media composed of RPMI with 5% HS, 1% FBS, 2 mM glutamine, and 1 x antibiotic antimycotic solution. Cells were then placed in 24 well-plates containing pretreated PVDF sheets and incubated overnight at 37 °C, 5% CO_2_ in order to allow cells to attach. For the following nine days, the cells were stimulated by ultrasound five times a day for 10 min within an ultrasonic bath (VWR USC300DF, nominal power and frequency: 80 W, 132 kHz) at 37 °C. The media was replaced daily.

For chemical stimulation experiments, PC12 cells were harvested and transferred to a PLL coated 24 well plate in differentiation media supplemented with 50 ng/mL neuronal growth factors (NGF). PC12 cells were maintained at 37 °C, 5% CO_2_ for nine days and the media was replaced daily.

Inhibitor experiments were conducted by supplementing differentiation media of piezoelectrically or chemically stimulated PC12 cells with 50 nM K252a, 50 μM lanthanum (III) chloride (LaCl_3_), or 10 μM roscovitine. Media was exchanged daily.

### Imaging

In general, all bright field and fluorescence images were obtained using an inverted optical microscope (Olympus IX-81).

Prior to imaging, cell media was removed, the cells were washed three times with PBS, and fixed in 4% paraformaldehyde solution for 15 min. Then, the cells were permeabilized with 0.1% Triton – X for 10 min. For tubulin staining, the cells were incubated with monoclonal antibody to β3 – tubulin for 3 h at room temperature and, subsequently, labeled with goat-anti-mouse secondary antibody conjugated with Alexa Fluor® 568. Cells were washed three times before 2 μg/mL Hoechst 333452 solution was added. Afterwards, cells were washed again with PBS three times and used for imaging.

For SEM images, PC12 cells differentiated on PVDF membrane were fixed for 15 min in 4% paraformaldehyde. The fixed cells were washed three times in DI water and placed in 1-butyl-3-methylimidazolium tetrafluoroborate for 30 s. The cells were then washed in a container with DI water for 30 sec and air dried, before imaging with SEM (Zeiss Ultra) operating at 3 kV.

### Data analysis

The quantitative results of neurite outgrowth were obtained by analyzing microscopy images in ImageJ (National Institute of Health, USA) using the NeuronJ plug-in software. All quantitative measurements are expressed as mean values, and error bars indicate the standard deviation of the mean. Statistical comparison of neurite length under different stimulation conditions was conducted by using the non-parametric Mann-Whitney U test.

## Electronic supplementary material

Supplementary Information
