# Supplementary material for: Ultrasound-mediated piezoelectric differentiation of neuron-like PC12 cells on PVDF membranes
Source: Sci Rep. 2017 Jun 22;7:4028. doi: 10.1038/s41598-017-03992-3 (PMC5481323; doi:10.1038/s41598-017-03992-3)
Supplement: Supplementary file 1 — Supplementary Information [file 41598_2017_3992_MOESM1_ESM.doc]

**Supplementary Information For**

Ultrasound-mediated piezoelectric differentiation of neuron-like PC12 cells on PVDF membranes

Marcus Hoop, Xiang-Zhong Chen*, Aldo Ferrari*, Fajer Mushtaq, Gagik Ghazaryan, Theo A. Tervoort, Dimos Poulikakos, Bradley J. Nelson, and Salvador Pané*

|  |  |
| --- | --- |

**Fig. S1** XRD pattern (left) and Infrared spectrum (right) of α- and β- PVDF membranes.

In Fig. S1, the XRD patterns of α- and β- PVDF are presented. For β-PVDF, the characteristic diffraction peak at 20.6°, which represents the superimposed diffraction peaks of (110) and (200) crystal plane, is clearly observed. No other peaks can be detected. For α-PVDF films, only the characteristic diffraction peaks at 17.8° (100), 18.5° (020), 20.0° (110) and 26.5° (021) are observed, which indicates high purity of α phase. Similar conclusions can also be drawn from the infrared spectra (Fig. S1, right). While the peaks at 840 cm^–1^(CH_2_ rocking) and 1274 cm^-1^ corresponding to β-phase are observed in the β-PVDF film, the characteristic absorption bands at 613 and 763 cm^-1^ (CF_2_ bending and skeletal bending), 795 (CH_2_ rocking) and 975 cm^-1^ (CH_2_ twisting) representing α phase are found in the new α-PVDF film. A very small absorbance band at 840 cm^-1^ can be observed in this film as well. According to literature (Martins P., et al., Progress in Polymer Science, 2014, 39, pp 683–706), the fraction of β phase *F(β)* can be determined by


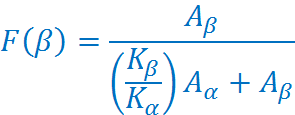


where A_α_ and A_β_ are the absorbance bands at 763 and 840 cm^−1^; K_α_ and K_β_ are the absorption coefficients at the respective wavenumber, which values are 6.1 × 10^4^ and 7.7 × 10^4^ cm^2^ mol^−1^, respectively. After baseline correction of each absorption peak, vaues of A_α_=0.3832, A_β_=0.0349 are obtained. Therefore, *F(β)* is about 6.5 %.


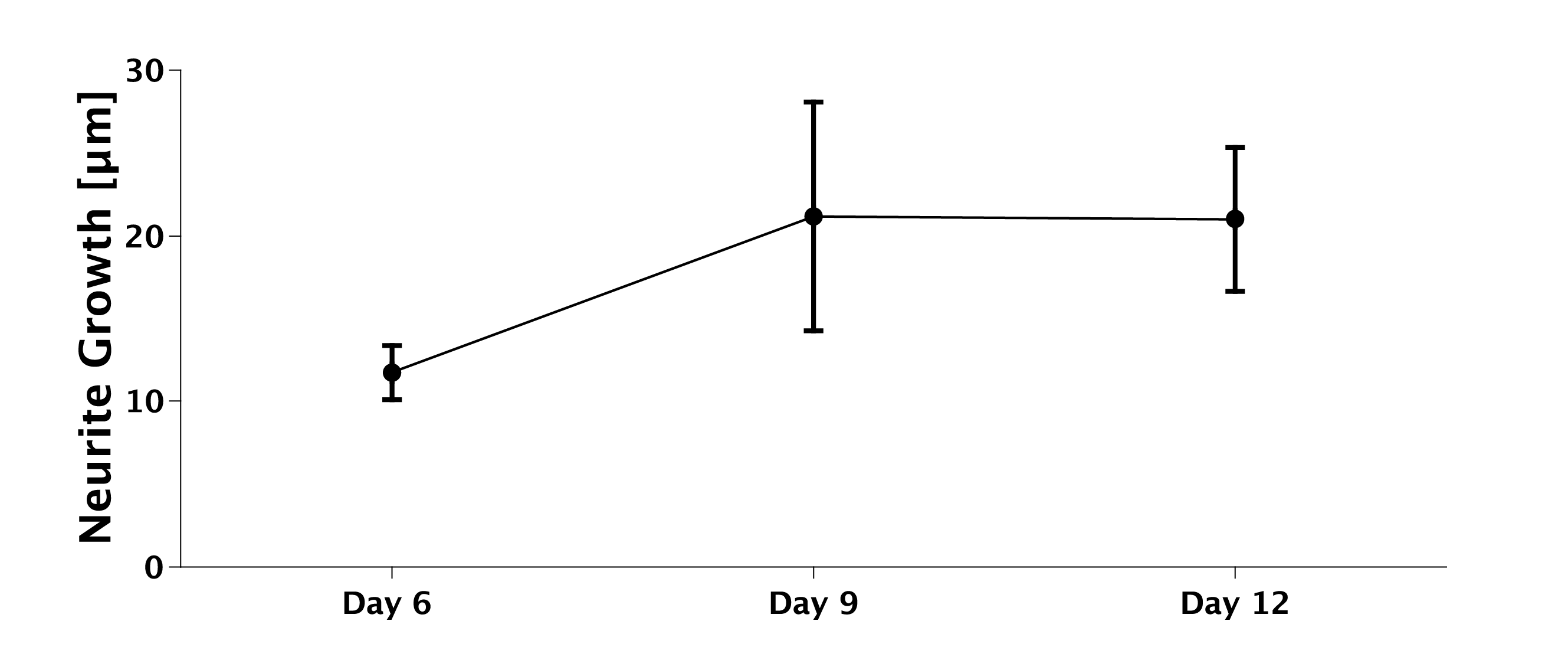


**Fig. S2** Development of neurite outgrowth upon US stimulation of differentiated PC12 cells.


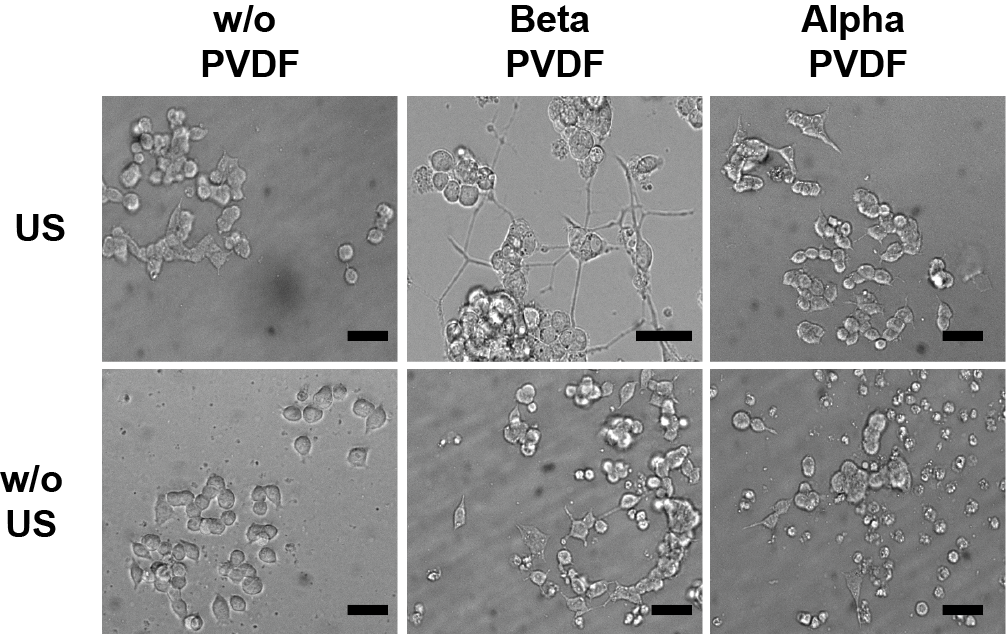


**Fig. S3** Optical image of PC-12 cells on well plate (left), β-PVDF (middle) and α-PVDF (right) with (upper row) and without (lower row) ultrasound stimulation. Scale bars in the images are 20 μm.

**Fig. S4** DSC traces of α- and β-PVDF.

Fig. S4 shows DSC (Differential scanning calorimetry) traces of both α- and β- PVDF. The crystallinity can be derived from the melting enthalpy. Assuming 93.07 and 103.4 J g^-1^ are the melting enthalpies of fully crystallized α- and β- PVDF, (R. Gonçalves, P. Martins, D. M. Correia, et al., RSC Adv., 2015, 5, 35852), the crystallinity for our α- and β- sample is 44.3% and 50.0%, respectively, calculated according to the equation:


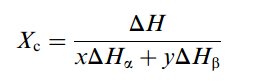


where x and y indicate the fraction of α and β phase present in the sample (calculated from IR). As the crystallinity of these two films are similar, it is unlikely that differences in cell behavior can be explained in terms of crystallinity differences.


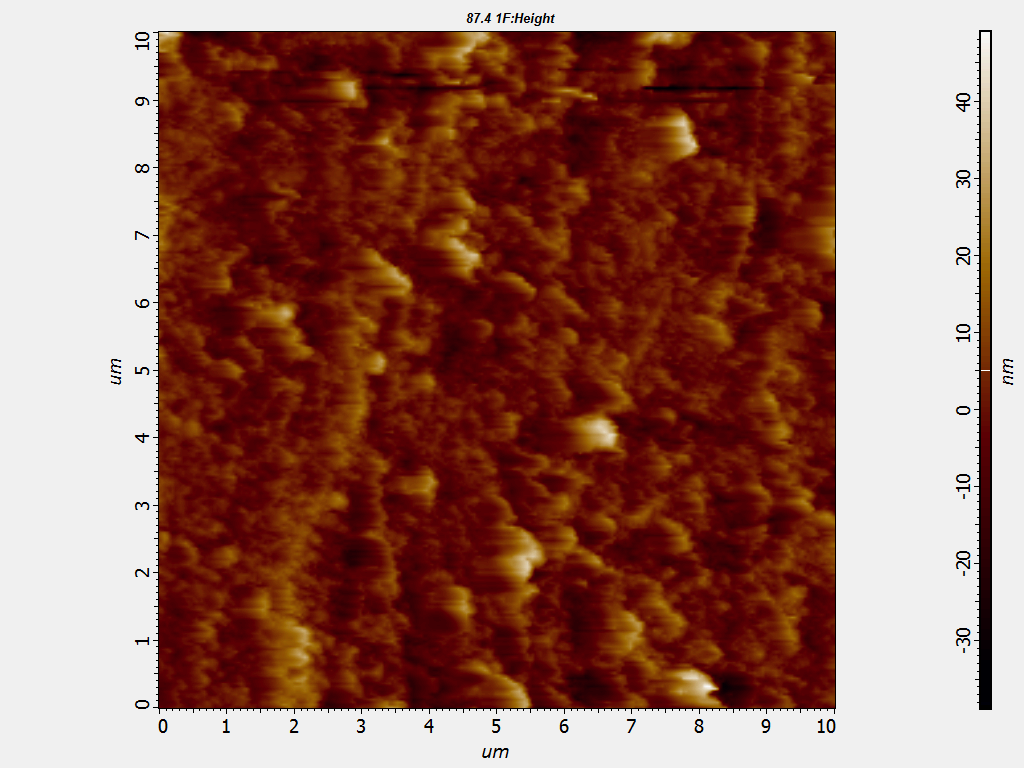

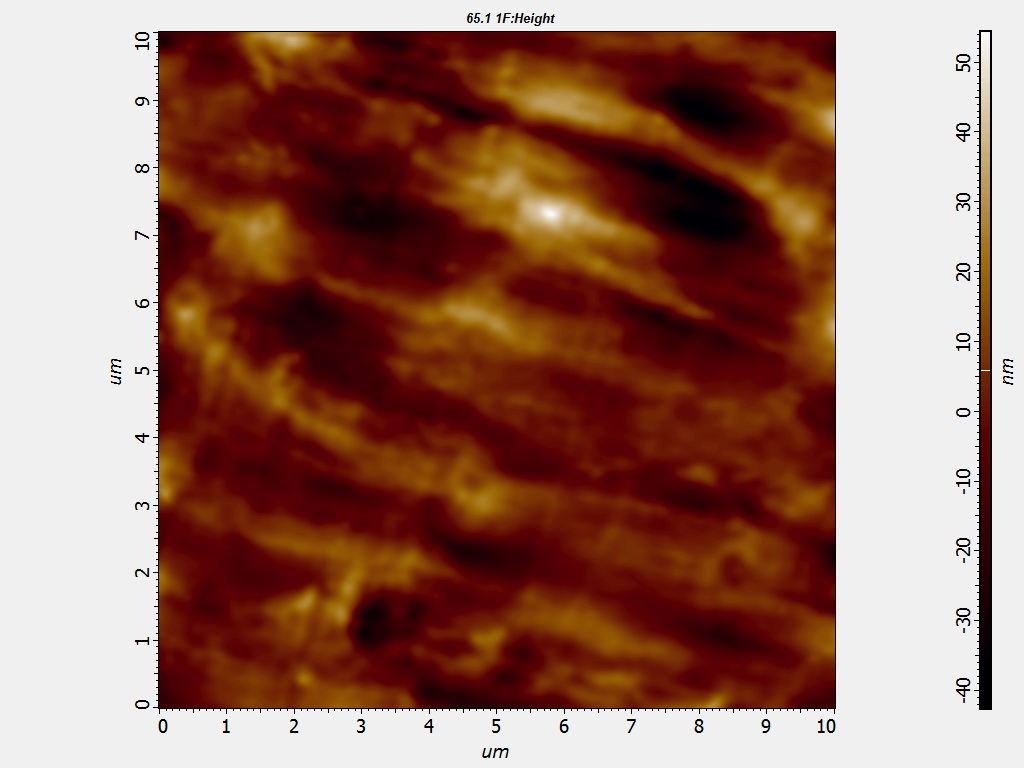


**Fig. S5** Topographic image of α-PVDF (left) and β-PVDF (right). The root mean squares for α-PVDF and β-PVDF are 7.418 nm and 11.807 nm, respectively.


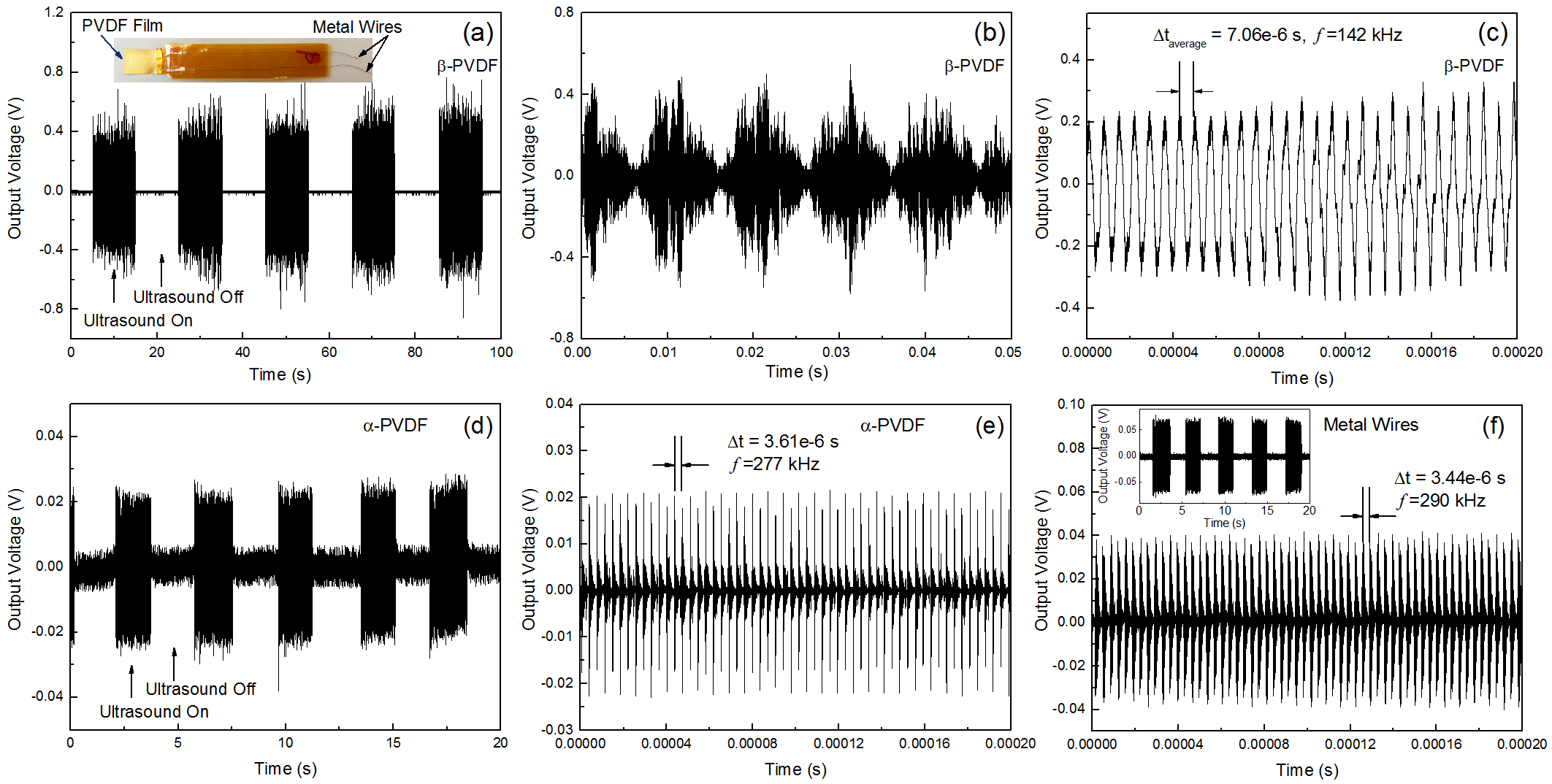
**Fig. S6**. The output voltage of PVDF films and control samples under ultrasonic stimulation. (a)-(c) are the recorded voltage signals generated by β-PVDF sample under water at time intervals of 100 s, 0.05 s and 0.0002 s, respectively. The inset in (a) is a picture of a typical sample. (d) and (e) are the signals recorded from an α-PVDF sample under ultrasonic stimulation in water bath at time intervals of 20 s and 0.0002 s, respectively. (f) shows the signal recorded from a pair of metal wires immersed in water bath under ultrasonic stimulation. The time interval is 0.0002 s. The inset shows the signal recorded in 20s.

As shown in fig. S6, the voltage output generated by piezoelectric β-PVDF matches well with the on/off status of ultrasound. The transient voltage can be higher than 400 mV (Fig. S6a). Fig. S6b shows the output voltage during a time interval of 0.05 s. The voltage generated during the ultrasound stimulation is not constant but fluctuating. In Fig. S6c, the signal in an even shorter time interval (0.0002 s) is presented. It can be seen that the voltage output is periodical, and the period is about 7.06e-6 s, which corresponds to a resonance frequency of 142 kHz, a value close to the nominal frequency (132 kHz) of the ultrasound bath. For α-PVDF (Fig. S6d), only noise was observed. Although it seems that the voltage generation corresponds to the ON/OFF status of ultrasound, 3 evidences clearly state that these signals are not piezoelectric signals from the α-PVDF film. First, the maximum value of the signal is only about 20 mV, much smaller than that of the β-PVDF (400 mV); second, the resonance frequency of the signal is about 277 kHz (Fig. S6e), which is deviated far away from the nominal frequency of the ultrasound generator; third, and the most important, we did the control experiments by only inserting two metal wires into the water bath under the same stimulation conditions, and we got similar responses as what we got from α-PVDF sample, i.e. small amplitude (~50 mV) and very large frequency (290 kHz) (Fig. S6f). These results clearly state that the signal of the α-PVDF sample comes from the connecting circuit instead of piezoelectricity. Please note that during the cell stimulation process the films are not connected to metal wires and there is no electric voltage output generated by wires.

Table S1. Young’s Moduli of α- and β-PVDF.

|  | Measuring Angle ^a^ | α-PVDF | β-PVDF |
| --- | --- | --- | --- |
| Young’s Modulus | 0° | 1369 (MPa) | 2015 (MPa) |
|  | 90° | 853 (MPa) | 1511 (MPa) |

^a^ The measuring angle specifies the angle between the tensile test direction and the orientation (drawing/extrusion) direction of the films.
